# Predicting perceived level of disturbance of visitors due to crowding in protected areas

**DOI:** 10.1371/journal.pone.0197932

**Published:** 2018-06-13

**Authors:** Jasminka Klanjšček, Sunčana Geček, Nina Marn, Tarzan Legović, Tin Klanjšček

**Affiliations:** Division for Marine and Environmental Research, Rudjer Bošković Institute, Zagreb, Croatia; U.S. Geological Survey, UNITED STATES

## Abstract

Managing the disturbance of visitors due to crowding is an important management task in protected areas with high use levels. To achieve this, managers need to know how the use level affects the perceived disturbance due to crowding. Here we present a method to predict the level of disturbance as a function of use level measured by number of visitors. In contrast to the visual approach where subjects are asked to evaluate acceptability of use levels from manipulated images of scenery, our approach uses data gathered from actual experiences: actual (measured) use levels and concurrent on-site data on levels of disturbance experienced by visitors. Using the example of Nature Park Telašćica, we show how these data can be acquired with limited resources (a smart-phone and short, time-stamped questionnaires), and demonstrate the subsequent analysis and model fitting. The resulting model estimates the probability that a visitor experiencing a given use level will report certain level of disturbance. We suggest a way of using the probability density functions to define an inherent limit of acceptable disturbance (LAD) due to crowding; the LAD can also be set to a desired value by management. Regardless of the definition, LAD can be used to determine the maximum acceptable use level as dictated by crowding considerations. The method gives predictions consistent with previous literature and can be used even when data are collected at low use levels.

## Introduction

Protected areas (PA) play a key role in ecosystem health, but also serve as areas for recreation. A number of management frameworks were developed to balance nature protection with recreation [1–5]. These frameworks recognize the need to ensure quality of visitor experience, which may be degraded by crowding, defined as ‘subjective and negative judgement about given amount of visitor use’ [6]. Due to recent increases in ecological awareness and inclusion of PA visits into tourist packages, PA visitation rates and, therefore, importance of disturbance by crowding is likely to increase. Hence, properly estimating acceptable level of use is of increasing importance.

Research of crowding originated in the USA, focusing primarily on recreation in wilderness areas. The research uses reported encounter rates, and relies on two basic assumptions, i.e. that perceived crowding: (i) is a subjective evaluation of level of use differing from the objective (measured) level of use, and (ii) can be used as an indicator of the visitor-defined acceptable and/or desirable level of use. The quality standards ensuring the satisfactory visitor’s experience are then specified by combining the reported number of encounters, perceived crowding, and actual number of people in an area (e.g. [7–11]).

The relationship between actual (measured) density, reported number of encounters (a subjective measure of number of visitors in an area, i.e., perceived density), and perceived crowding has been extensively explored. There is a positive correlation between the actual and the perceived density, as well as a positive correlation between the actual density and the perceived crowding. However, the effects of *perceived* density on perceived crowding were found to be stronger and more consistent than effect of *actual* density on perceived crowding [12]. In addition, the evaluation of crowding is often influenced by the area (type of resource, encounter, and activity) as well as personal preferences (e.g. motivation of visitors) [7–9].

In high use areas visitors have difficulties in reliably estimating the number of encounters, so a visual approach is the suggested method of choice to determine crowding norms [13]. The visual approach uses image-based questionnaires, i.e. scenery shots with various number of people pasted into the shot, to estimate individual levels of acceptable crowding. From the individual levels, norm curves that can be used to determine overall acceptable use levels are constructed. The method is widely accepted and often used for general visitor management [14–17], as well as specific activities such as diving [18–20], kayaking [21], and nautical tourism [11].

However, the visual approach has potential issues. For example, the sequence of the image-based questionnaires can create bias [22]; a scenery shot to represent the whole area might be difficult to identify; and obtained results (acceptable use level) based on the scenery shot characterizes only that particular micro-locality, but may or may not characterize the whole locality. Additionally, the image does not convey other real-world conditions that might modify visitor experience; for example, environmental factors such as heat and noise might exacerbate effects of crowding, while other vistas available at the site might reduce them.

The in-situ approach presented here surveys actual use levels and concurrent on-site data on experienced disturbance levels using (simultaneously) time-stamped short questionnaires and visitor counting. The approach is consistent with previous approaches, and overcomes the issues outlined above. The consistency is ensured by incorporating the same basic concepts and assumptions, such as assuming that the perceived number of encounters is a subjective variable, and that visitors are on average more disturbed when visitation rate is higher. Although the results and variables of the new approach are comparable to those of previous approaches, the in-situ approach has a number of advantages. First, each participant evaluates perceived use and disturbance level only once, thus avoiding bias noted by Gibson *et al*. [22]. Second, the in-situ nature of our approach guarantees i) that the locality as a whole is evaluated rather than just the micro-locality captured by the scenery shot, and ii) that the environmental conditions are taken into the account. Each questionnaire is also time-stamped, thus making relating the objective evaluation (measured number of people) and the subjective evaluation (visitors’ evaluation of number of people and disturbance) possible. The relation can then be used to create a disturbance level model, from which the limit of acceptable disturbance (LAD) due to crowding is calculated as an equivalent of minimum acceptable condition derived from the norm curves. Finally, the required technical (e.g. image manipulation) skills are greatly reduced, and the process of identifying a representative scenery shot is eliminated.

We introduce and explain the method using as an example nature park Park prirode Telašćica (PP Telašćica)—a PA in Croatian coastal area visited by many individuals and groups.

## Methods

Methods include description of (i) study site and experimental setup, (ii) data gathering, handling and processing, (iii) statistical analysis, (iv) description of the predictive model, and (v) method for calculating maximal use level using the predictive model.

### Study site and experimental setup

PP Telašćica is one of 11 nature parks in Croatia, located on southern part of island Dugi otok and covering an area of 70.5 km2, 64% of which is sea. The area has been protected since 1980 because of its valuable natural and cultural heritage [23], and has received the status of a Nature park in 1988. The Park is near four larger coastal cities (Zadar, Zaton, Biograd, Vodice), which makes it an attractive offering as a part of organized one-day tourist boat excursions.

Visitors of the Park can roughly be classified into three groups: (i) nautical tourists [boaters / yachters], (ii) tourists on one-day excursion boats, and (iii) tourists lodging on the island. There is a pronounced seasonal, but also a strong daily variability in the number of Park visitors. Nautical tourists frequently visit from May to October, whereas the other two visitor groups mostly visit during July and August, resulting in a seasonal peak in visitor numbers during those two months. During July and August, the number of visitors peaks daily between 11:00 and 16:00 due to the boat excursions.

Telašćica bay (the biggest and safest natural harbor in the Adriatic), vertical sea cliffs Stene, and Salt lake Mir are three separate localities that make up the three main natural and tourist attractions in the Park. Consultations with the Park management identified the Salt lake Mir as the most likely locality to be subject to the detrimental effect of crowding for at least two reasons: i) it is a relatively small area visited by as much as 80% of Park visitors [24], and ii) visitors aggregate near the entrance into the area (which serves as a beach), even though there is an educational footpath around the lake. Visitors engage mainly in activities related to the beach area (resting, sun-bathing, and swimming), while activities connected to the footpath (walking, cycling, running) are secondary. During summer, water in the lake is often warmer than in the surrounding sea, making the lake attractive for swimming.

### Data gathering, handling and processing

**Fig 1:**
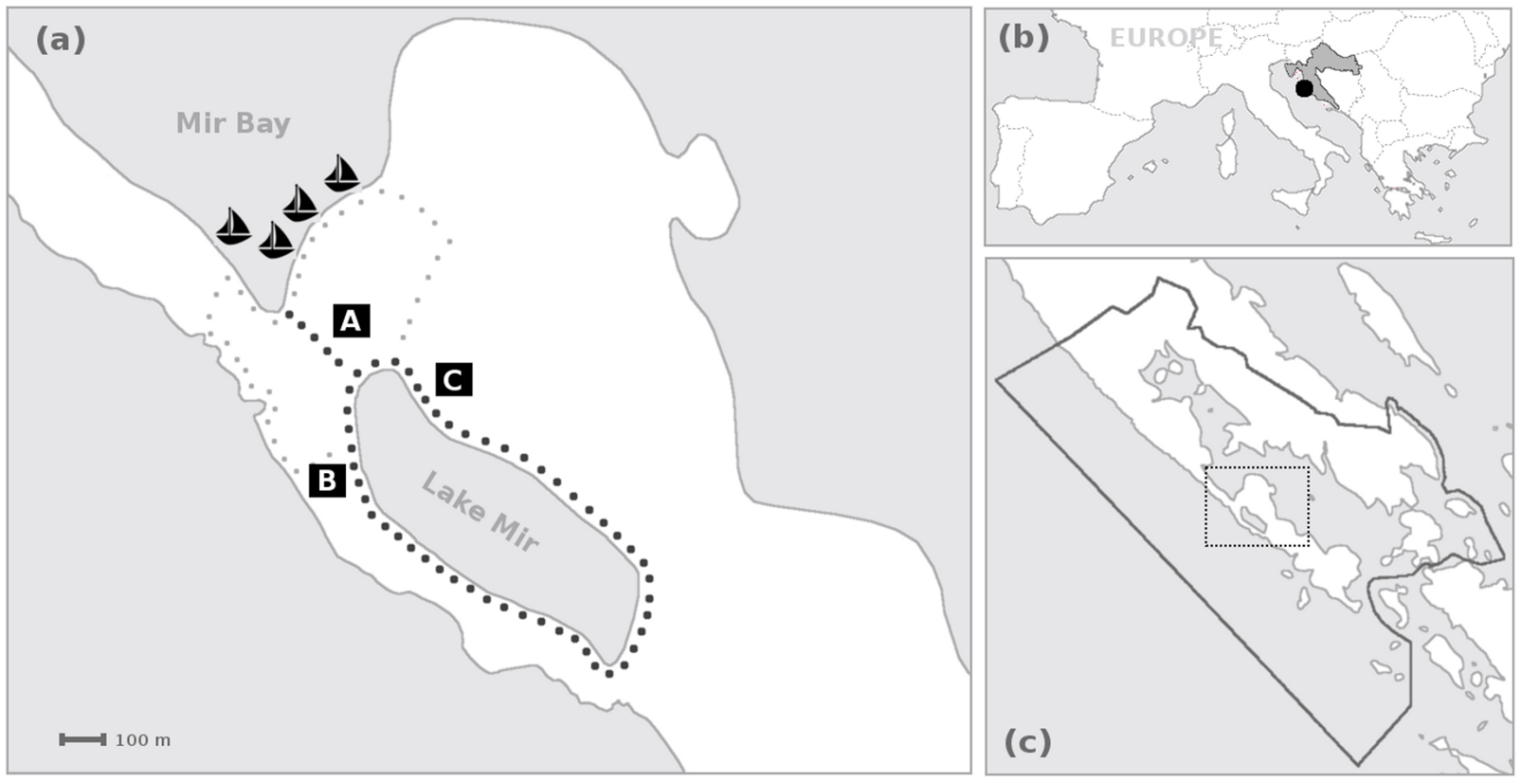
The locality: Salt Lake Mir. Panel (a): details of the locality and experimental setup. Boats indicate location of the excursion ship docks; black dots represent the main entrance to the Lake Mir locality and the footpath around the Lake Mir; gray dots represent small side-way entrances; lettered squares represent the survey locations; the area between the survey locations is the beach. Panel (b): PP Telašćica in the European and Croatian context. Panel (c): Dugi Otok island region. Solid line represents the PA boundary, and dotted line the area shown in panel (a).

Data collection included simultaneous counting of visitors in the area, and questioning a subset of visitors. To maximize likelihood of capturing peak use levels, experiments were timed to coincide with the peak of the tourist season in the area (late July—mid-August). Experiments were performed on 31^*st*^ of July and 13^*th*^ of August 2015, from 10:00 to 17:00. Data was gathered simultaneously on three locations within the Salt lake Mir locality (Fig 1). The location (A) is the main entrance to the lake Mir area, and the other two locations (B and C) are situated on the foot path around the lake (Fig 1).

*Number of visitors* in the area was tracked on three locations within the Salt lake Mir locality (Fig 1) by using an entry-exit counting method. Counting was performed using a simple Android smart-phone application specifically developed for the study, but any similar application would suffice. Each direction of possible tourist movement had one button assigned. Pressing any of the buttons in the application recorded the date, time and the name of the pressed button into a text file, thus documenting both time and direction of visitors’ movement. The application also allowed for direct text inputs (e.g. ‘35 people’). Upon the end of survey, the text file was sent via email for further analysis.

Data processing of observed visitor number included three main steps. First, the text file was processed to interpret the button code (e.g. interpret ‘1.1’ and ‘2.1’ as respectively ‘entry’ and ‘exit’). Each ‘entry’ increased, and each ‘exit’ decreased the number of visitors in the area by one. Possible text comments made by person using the application were cleaned up and resolved manually. Second, the files were imported into Matlab and entries and exits from all three locations were summed up into one minute intervals. To account for two small sideways entries (see Fig 1), the number of entries in the main entrance was proportionally increased (by 5.8%) to match the recorded total number of excess visitors (71 during the first, and 117 visitors during the second day of the experiment). Third, the number of people in the Salt lake Mir locality (the beach and foot path) at any given time was calculated by subtracting the exits from entries (up to that time) and adding the initial number of people present on the footpath or beach before the counting started. The initial number of people on the first day was estimated as 20 on beach and 5 on footpath, and on the second day it was estimated as 17 on beach and 3 on footpath.

The same locations (A, B, and C in Fig 1) were used to collect *time-stamped mini-questionnaires*. The questionnaires were composed of three statements/questions, which could be responded to by using nine-point scale, and included an “I don’t know” option. Each surveyed visitor was asked to evaluate the number of visitors in the area, perceived disturbance, and overall satisfaction. The statements/questions were formulated as follows:

1. *The number of visitors at this location (Salt Lake Mir) is*: The provided 1-9 scale beneath the statement had 1 noted as *Extremely small*, and 9 as *Extremely large*.
2. *To what extent does the number of visitors at this location (Salt Lake Mir) bother you?* The provided 1-9 scale beneath the question had 1 as *Not at all* and 9 noted as *Extremely*.
3. *How satisfied are you with your visit to this location (Salt Lake Mir)?* The provided -4 to +4 scale beneath the question had -4 noted as *Extremely dissatisfied*, 0 as *Neutral*, and 4 as *Extremely satisfied*.

The questionnaires were available in 11 different languages (Czech, Croatian, English, French, German, Hungarian, Italian, Polish, Slovak, Slovenian, and Spanish), reflecting the very diverse nationality structure of Park visitors. Most of the visitors could answer the questionnaire in their native tongue, which we hoped would contribute to lower rejection rate in survey participation.

Visitors exiting the Salt Lake Mir locality through survey location A, and entering the beach area through survey locations B and C were asked if they were willing to answer a short questionnaire. If they accepted, they would fill out the questionnaire by themselves, and the interviewer time-stamped the questionnaire. After that, the next visitor encountered is asked to participate. When a larger group was approaching, one person was randomly selected to answer the questions. In case of continuous flows of visitors (that were not in a group), each tenth visitor was stopped and asked to participate.

Questionnaires with the response to at least one of the three questions were included in further analysis. Some questionnaires had a fourth question (*How long did you swim?* or *How long did you walk?*). The fourth question was introduced for a pilot study, so the answers were not analyzed for the purposes of this study.

#### Ethics and privacy considerations

The visitor survey was conducted as part of the project ‘Assessing carrying capacity for tourists in nature protected areas’ approved by Croatian Science Foundation and implemented by Rudjer Bošković Institute. The field permit was issued by Ministry of Environmental and Nature Protection, Croatia (Class UP/I-612-07/15-33/04, No:517-07-2-1-1-15-6). The survey was designed and conducted in such a way that participants gave oral consent and answer the questionnaire voluntarily. The participant anonymity was preserved, and no personal data were collected. Observation of visitor number was done only by counting, without using any additional means (such as photographing, or GPS tracking) that could lead to a violation of participant’s anonymity.

### Statistical analysis

Since the questionnaires and measurements of visitor numbers were simultaneous, number of visitors at the locality at the time of interview were known. Hence, a matrix with the following four-variables could be constructed: (i) measured number of visitors on the locality (use level), (ii) evaluated number of visitors (perceived use level), (iii) evaluated disturbance, and (iv) overall satisfaction.

*Spearman’s rank correlation coefficient* (*ρ*) was used to determine a strength of association between each of two variables, i.e. to determine if there is a statistically significant relationship between each pair of variables. The data were verified to satisfy Spearman’s correlation assumptions (the data were at least ordinal, and the scores on one variable are monotonically related to the other variable). The rank correlation coefficient was calculated using Matlab2013 inbuilt Statistic toolbox.

### Predictive model of disturbance due to crowding

Predictive model of disturbance due to crowding was created by using ordinal logistic regression to describe the relationship between measured use level, *X*, and perceived level of disturbance, *Y*. To increase significance levels, the original nine categories of disturbance level data were grouped into five groups by combining similar levels of disturbances: minimal disturbance level (1 and 2) into group 1, low disturbance level (3 and 4) into group 2, moderate disturbance level (6 and 7) into group 4 and high disturbance level (8 and 9) into group 5. The neutral disturbance level (5) represented a center, and constituted group 3 that could not be combined with either low or high disturbance levels. The following ordinal logistic regression (cumulative link model) was fitted:

$$\begin{array}{c} \text{logit}\;\left(P\left(Y\leq j\right)\right)=\theta_{j}-\beta \left(X\right),\;j=1,\ldots ,J-1 \end{array}$$ (1)

where *θ*_*j*_ is the intercept of the *j*^*th*^ cumulative logit, and *β* is the regression coefficient for a predictor variable (use level, *X*). The use level (*X*) represents the number of visitors on the locality expressed in hundreds, and *j* = 1,…, *J* − 1 is the index of the group (*J* = 5).

The predictive model was fitted by the maximum likelihood method implemented in clm function of *Ordinal package* [25] to obtain coefficients *θ*_*j*_ and *β* with confidence intervals, standard error, z-test, and the associated p-values.

The ordinal logistic regression is built upon the assumption that the effect of the predictor variable (*X*) is the same for all logit functions. Hence, appropriateness of the use of logistic ordinary regression was verified by testing the assumption on proportional odds (parallel logits) underlying the regression. Likelihood-ratio test was used to test against the null hypothesis that there is no difference between model with the single scaling parameter (*β*), compared to the model with different parameters across logits (*β*_*j*_, *j* = 1,…,*J* − 1). In addition, the full model was tested against the global null hypothesis (*β* = 0). All calculations were performed using *Ordinal package* [25] in R Software.

### Predictive model of disturbance to calculate limit of acceptable disturbance

The limit of acceptable disturbance (LAD) due to crowding is the maximum acceptable level of disturbance. Our model predicts probability that a person is disturbed by crowding to a certain degree (on a scale 1-5) as a function of use level. Conversely, we can set a desired degree of disturbance, and look up the corresponding use level. The maximum acceptable use level is, therefore, the use level corresponding to the LAD. Although the LAD can be arbitrarily set, we suggest a minimal LAD defined as the point where a person is equally likely to experience low and high disturbance level. Mathematically, this point can be found at the intersect of the probability curve of low level disturbance (groups 1 and 2), and the probability curve for high disturbance (groups 4 and 5).

Note that the model, once fitted, can predict levels of disturbance even for use levels higher than the maximum use level used to fit the model. Therefore, the approach can be used to determine maximum use levels (as limited by crowding) even for areas that have not reached such high use levels.

## Results

### Collected short questionnaires

In total, 362 questionnaires were collected, of which 356 were time stamped, and 344 had at least one of the three question answered. On July 31^*st*^, 77 questionnaires were collected from visitors exiting the beach (location A), and 44 from visitors exiting the footpath (locations B and C), making the total of 121. On August 13^*th*^, a total of 223 questionnaires were collected (166 from the beach exit, 57 from the footpath exit). On both days, when compared to the measured number of people that visited the site, at least 10% of the visitors exiting the locality were surveyed.

**Fig 2:**
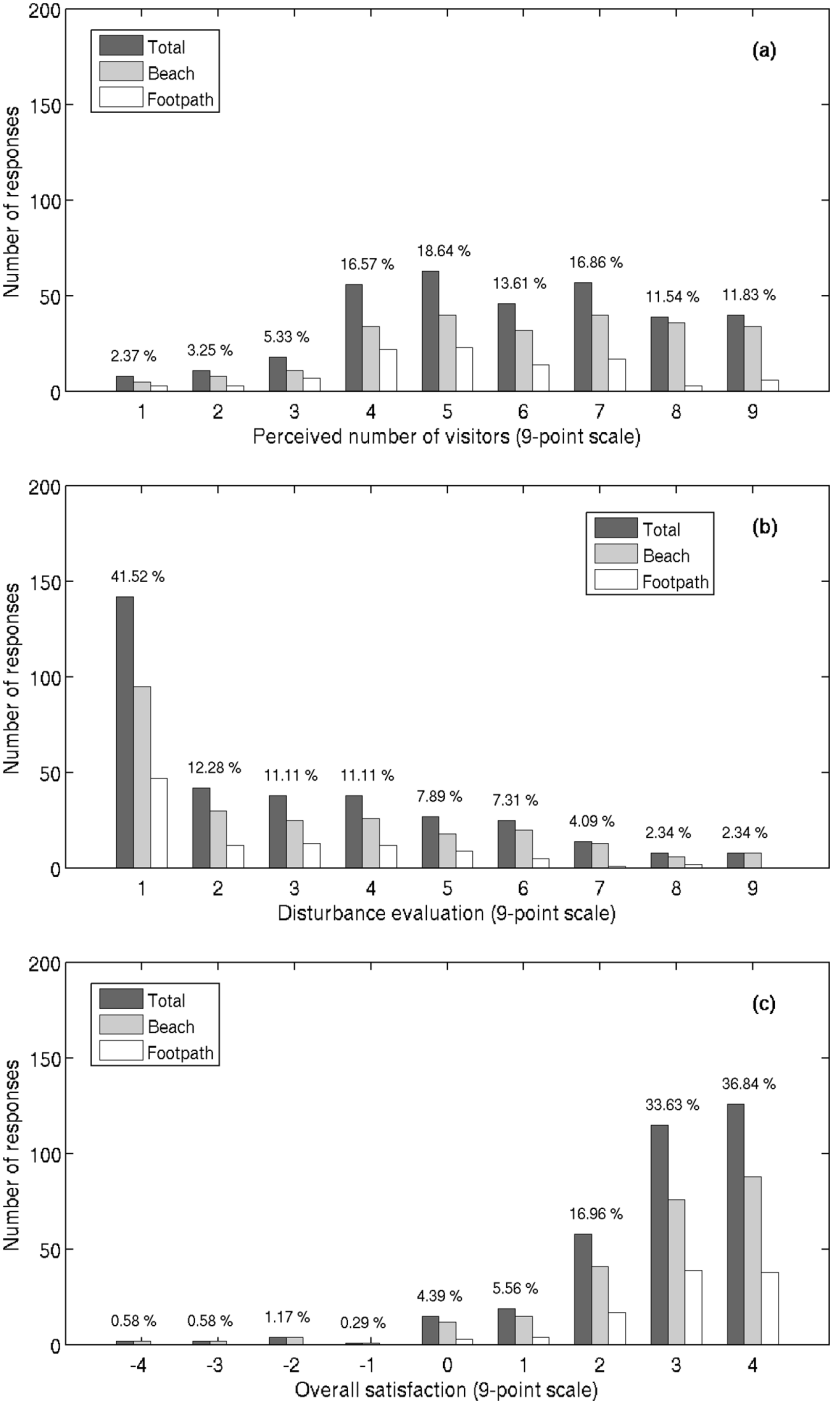
Frequencies of visitors’ responses collected on the Jezero Mir locality. Frequencies are related to: a) perceived number of visitors on the scale from 1 (extremely small) to 9 (extremely large), b) disturbance level on the scale to 1 (not at all) to 9 (extremely) and c) overall satisfaction on the scale from -4 (extremely dissatisfied) to +4 (extremely satisfied). All results are presented as joint responses as well as distinctive regarding the location of exit survey (beach or footpath). Percentage for each response category are shown for joint responses only.

A slight majority (54%) of visitors feel that perceived level of use (number of people) in the locality is large, giving a rating of 6 and higher on scale from 1 (extremely small) to 9 (extremely large) (Fig 2(a)). The average perceived use level was 5.8, with the visitors at footpath exits (locations B and C) reporting slightly lower average of 5.2. High perceived use levels were reported by 41% of the visitors on the footpath, and 59% of the visitors on the beach (average grade of 6.1). Despite the relatively high perceived use level, only 16% of the visitors are disturbed by crowding (report disturbance level of 6 or higher) (Fig 2(b)). As even more than 41% of the visitor rated the disturbance level as ‘low’ or ‘non-existing’ (grades 1 or 2), the overall disturbance level is low, with the average of 3. Again, the disturbance level on the footpath is lower than the one recorded for the beach: only 8% of the visitors on the footpath are disturbed (grade 6 or higher), compared to 20% on the beach (average grade 3.1).

A large majority of visitors are satisfied with the visit to the locality (Fig 2(c)). On the scale from -4 (extremely dissatisfied) to +4 (extremely satisfied), the average rating was 2.8, with 93% of visitors responding positively (1 or higher). Satisfaction level for the visitors on the footpath is even higher: 97% of the visitors are satisfied, and the average rating is 3. None of the visitors on the footpath were dissatisfied (grade zero or less). A large majority of beach visitors (91%) were satisfied, with the average grade of 2.7.

### Daily visitor number variation

**Fig 3:**
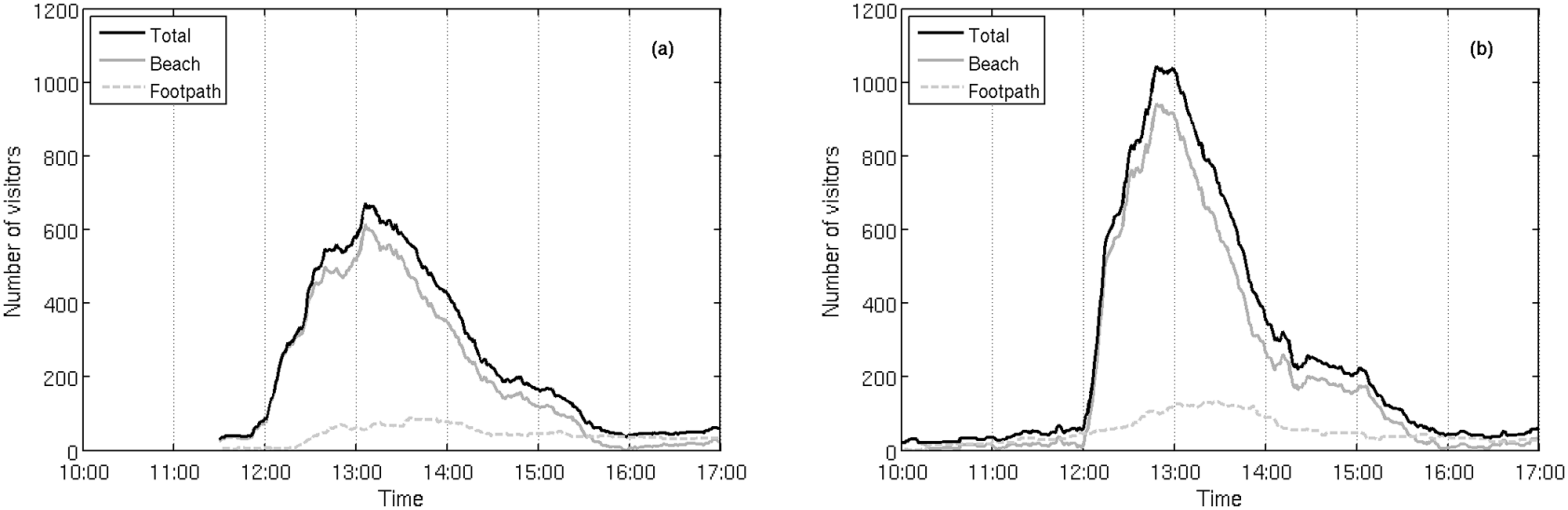
Diurnal variation of visitor number on the locality Salt Lake Mir. Variation on the locality (beach and the surrounding footpath) was observed during high season in summer on two days: (a) Jul, 31^*st*^ 2015, and (b) Aug, 13^*th*^ 2015. Delineated residing number of visitors on site has been constructed using visitor entry and exit data in one minute intervals. Gray solid line represents residing number of visitors on the beach area, gray dashed line residing number of visitors on footpath, and black solid line total residing number of visitors on the locality.

Visitor number on the locality (Salt Lake Mir) strongly varies during a single day (Fig 3). On July 31^*st*^, the locality had a total of 1299 visitors, with a peak of 672 visitors at (close to) 13:00 hours. On August 13^*th*^, the total was 2113, with a peak of 1044 at about the same time (13:00 hours). The intensive visitor pressure occurs in the period between 12:00 and 16:00 hours, mirroring the times of excursion ship stay in the Park.

Most of the people entering the locality visit only the beach: only 206 visitors (16%) entered the footpath on July 31^*st*^, and only 271 (13%) visitors on August 13^*th*^. Although the total number of visitors is higher during the second day, the visitation patterns are rather similar for both days. As most visitors stay on the beach, the maximal observed number of visitors at the beach are close to the total number of visitors at the whole locality, 615 (92%) on July 31^*st*^, and 942 (90%) on August 13^*th*^. The maximal observed number of visitors on the footpath occurs approximately half an hour later than the on the beach area, and is 89 and 132 for the two days, respectively.

### Correlation among variables

Strength of association between four variables (level of use, perceived number of visitors, disturbance level and overall satisfaction) is significant (Spearman’s rank correlation) in four out of six possible cases (Table 1). Visitors who perceived higher use levels are also reporting higher disturbance levels (*ρ* = 0.3). Furthermore, the visitors who are experiencing higher disturbance level are also reporting lower levels of overall satisfaction (negative correlation; *ρ* = -0.2). However, no significant correlation has been found between overall satisfaction and use level, neither for the actual use levels (from counting) nor the perceived use levels (subjective, from questionnaires). The lack of correlation, which was expected to be negative, is partly related to weak strength of association among other variables. The results also support the hypothesis that the visitors will perceive higher use level as the actual use level increases (*ρ* = 0.36). In addition, higher actual use level leads to higher disturbance level (*ρ* = 0.29). The positive correlations of disturbance level and the actual use level make the construction of the predictive model possible.

**Table 1:** Strength of association between variables. The strength of association between level of use, perceived number of visitors, disturbance level and overall satisfaction was determined by the Spearman’s rank correlation coefficient (*ρ*). For each pair of variables *ρ* values, number of paired data (df), and significance level (p-value) are reported. Not significant (ns) association between variables are additionally noted.

	*ρ* = cor	df	p-value
Perceived use level vs Disturbance level	0.305	336	1.102*10^−08^
Perceived use level vs Overall satisfaction	0.027	336	0.628 (ns)
Disturbance level vs Overall satisfaction	-0.205	340	1.434*10^−04^
Use level vs Perceived use level	0.360	333	1.208*10^−11^
Use level vs Disturbance level	0.288	337	7.242*10^−08^
Use level vs Overall satisfaction	-0.073	337	0.179 (ns)

### Disturbance level in response to use level

Disturbance level was further analyzed in response to use level observed in the park using (i) absolute response frequency, (ii) disturbance level model, and (iii) calculating the LAD.

#### Frequency of reported disturbance levels for a range of use levels

**Fig 4:**
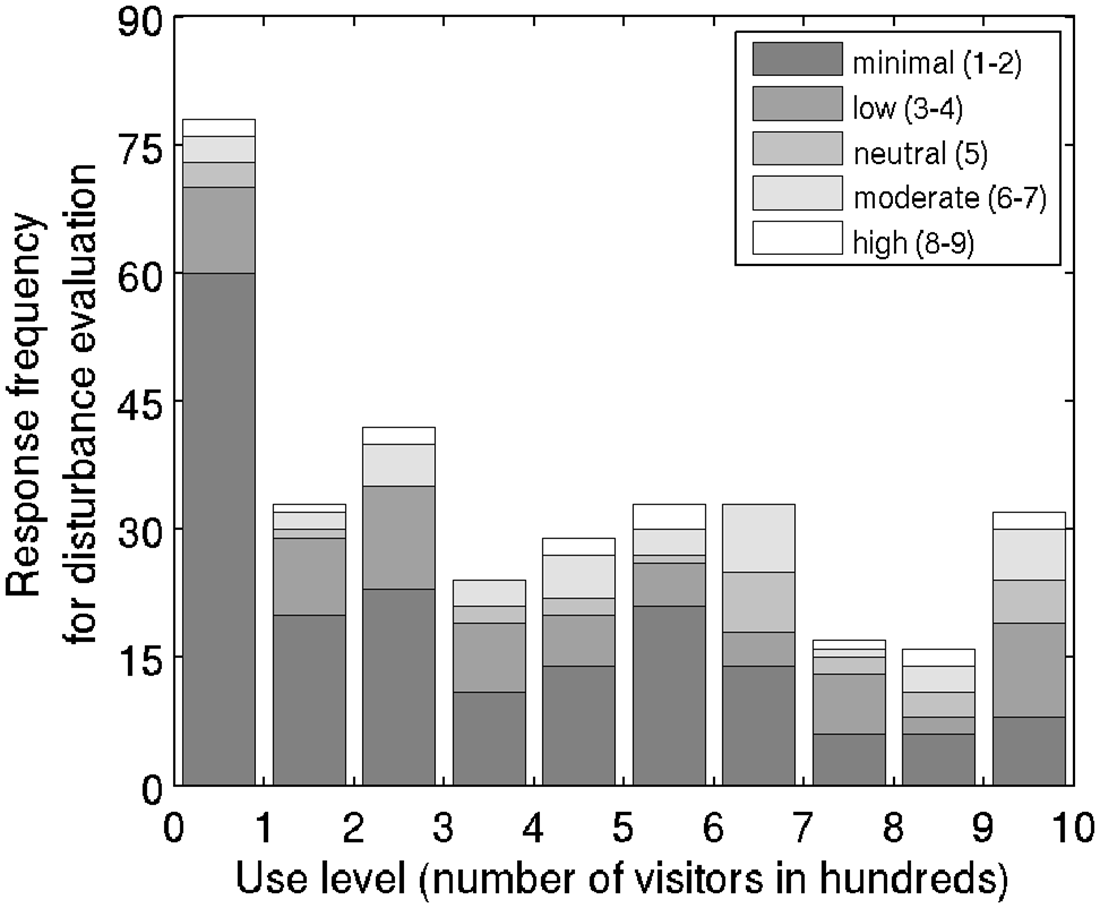
Measured visitors’ disturbance level in response to the observed level of use. Disturbance levels are shown in absolute frequencies and grouped by combining similar levels of disturbance (see Methods for details).

Frequency of reported disturbance is consistent with positive rank correlation (*ρ* = 0.288) between level of use and disturbance level (Fig 4). At low use levels, frequencies of low disturbance reporting are expectedly high—more than 50% of respondents report disturbance level of 1. Surprisingly, the low disturbance levels are reported for all use levels. However, as use level increases, the proportion of low disturbance level reporting decreases, and the incidence of highest disturbance level reporting increases.

The number of questionnaires representing each use level category is skewed. The lowest use level is also the most represented one (78 questionnaires collected) because it had the longest duration (until 12:00, and after 16:00). Other use level categories (>100 visitors) were more evenly represented (mean = 29, min = 16, and max = 42). Therefore, the number of recorded responses for each use level was sufficient to fit the disturbance level model.

Coefficients of the *predictive model for disturbance due to crowding* given by Eq (1) have significant p-values (Table 2). The likelihood-ratio test against the global null hypothesis (*β* = 0) proved that the use level (*X*) is a significant explanatory variable for disturbance level (*χ*^2^(1) = 30.6, p = 3.2*10^−8^). The assumption of proportional odds underlying ordinal logistic regression was verified as well: the hypothesis that the relationship between each pair of outcome groups is the same could not be rejected (*χ*^2^(3) = 3.32, p = 0.34). Therefore, the use of a single *β* is appropriate.

**Table 2:** Estimates of coefficients for prediction model given by Eq (1). First column: coefficient name. Second column: estimated coefficient values. Columns 3-6: standard error, lower 95% confidence interval, upper 95% confidence interval, z-value, and p-value. Coefficient *θ*_*j*_ (rows 1-4) and *β* regression coefficient (row 5) were estimated by fitting the model to use levels expressed in hundreds of visitors. The statistical significance tests (z-values and the associated p-values) indicate high significance (p < 0.001).

	Estimate	Std. Error	lower CI	upper CI	z value	Pr(> | *z* |)
*θ*_1_*	0.9415882	0.18483981	0.5793088	1.3038675	5.094077	3.504449e-07
*θ*_2_*	2.0240569	0.21234230	1.6078737	2.4402402	9.532048	1.542110e-21
*θ*_3_*	2.5423831	0.23024654	2.0911082	2.9936580	11.042004	2.396288e-28
*θ*_4_*	3.9824186	0.32161371	3.3520673	4.6127699	12.382615	3.245934e-35
*β*	0.1848905	0.03399295	0.1182656	0.2515155	5.439085	5.355501e-08

*Disturbance level data set was reduced to 5 groups

The *β* coefficient for use level is positive, indicating that the likelihood of a high disturbance rating increases with use level, i.e. that higher use level increases disturbance level of the visitors. For example, should the use level increase by 100 visitors (a unit change in use level *X*), the odds of a higher response *P*(*Y* > *j*) versus *P*(*Y* ≤ *j*) increase by a factor *e*^*β*^ = 1.2031, for every *j* = 1,…,*J* − 1.

Although model coefficients have significant p-values, low pseudo-R^2^ values indicate low strength of the association between disturbance level and use level (McFadden R^2^ = 0.036, Cox and Snell R^2^ = 0.087, and Nagelkerke R^2^ = 0.095). We attribute the low apparent association strength to the fact that frequency of responses citing low disturbance level is high for all measured use levels, and variation among individual responses to similar conditions is very high (Fig 4).

#### Using predictive model to determine LAD

**Fig 5:**
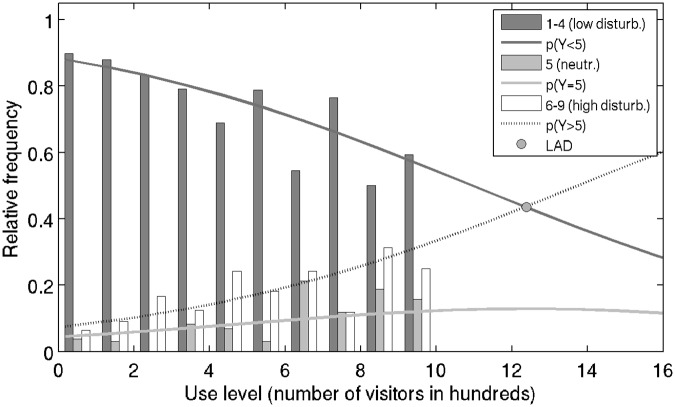
Predictive model of disturbance by crowding. Lines: predicted frequencies of disturbance level reporting as a function of use level. Disturbance levels are pooled into three categories (low, average, and high—see Methods for details). Solid black line: probability of reporting low disturbance (grade 1-4). Dotted line: probability of reporting high disturbance (grade 6-9). Solid gray line: probability of reporting neutral disturbance (grade 5), not used in determining LAD and maximum acceptable use level. Bars: relative frequencies of data pooled into three visitor disturbance level categories presented by the model. Intersection of the solid and dotted lines (gray circle) represents a point where the probabilities of being and not being disturbed are equal, and is used to calculate LAD and the corresponding maximum acceptable use level.

The limit of acceptable disturbance (LAD) due to crowding for the Salt Lake Mir locality is projected to be at 43% visitors reporting low (1-4) or high (6-9) disturbance levels, and 14% reporting neutral disturbance level (Fig 5). The locality is, therefore, predicted to support maximum acceptable simultaneous use level of 1238 visitors, which is higher than the currently observed peak use level of 1044. Since the current measurements suggest that number of daily visits to the locality is approximately equal to twice the peak use level, the results suggest that, under current visitation patterns, the maximal number of daily visits to the area should be 2470.

## Discussion

### Obtained results from study site to other crowding studies

The main relationships between variables obtained in our research are consistent with the general trends reported in the field of crowding research: the visitors will perceive higher visitor numbers as the real use level increases, higher perceived number will lead to higher disturbance level. We did not find significant correlation between (real or perceived) use level and overall satisfaction. We believe this is because the maximum acceptable use level has not been reached during our survey and, therefore, the overall satisfaction was not significantly reduced. The correlation between the disturbance level and the overall satisfaction is, however, significant (*ρ* = -0.20, Table 1), and on the higher end of the range (from -0.12 to -0.20) reported by Graefe *et al*. [12].

The results are somewhat clouded by high variability of individual responses. This is a common issue in crowding research. For example, Manning *et al*. [13] report standard errors of mean acceptance level across different use levels from 1.8 to 2.3. More interestingly, even the zero use level has a very high variability of acceptance level (std 2.3 for mean of 3.0). Nevertheless, the trends are clearly visible; this is why Manning *et al*. [13] could construct norm curves, and our model has significant coefficients.

### Possible method modifications

Our method is general even though our presentation is tailored to a specific locality. The crucial elements of our method are data on a range of use levels, and the corresponding disturbance levels. Other elements, such as the way of obtaining use levels, grouping of disturbance levels, and the definition of LAD, can be modified.

The number of visitors could be obtained in various alternative ways [26], such as direct on-site observing of the visitors by staff, counting visitors from image time series, or using on-site counter devices. Any of these would be appropriate for our method, as long as the the crucial elements are obtained. For example, locality has to be clearly defined, and the counting method has to account for all visitors; also, as many as possible disturbance levels have to be recorded for as great of a range (and variability) of use levels as possible. Therefore, knowing the visitation patterns for the location prior to the design of the experiment is helpful and may be necessary.

We measured the level of disturbance due to crowding by asking how much visitors were bothered by the number of people at the locality, but alternative questions could have been asked, e.g. to ask for level of crowding at the time of their visit as in Heberlein and Vaske [27]. We decided to measure the disturbance level instead of perceived crowding to avoid possible misinterpretations of the term ‘crowding’ by non-native English speakers, and/or possibile misinterpretations during translation to other 10 languages used in our study.

We recorded visitor disturbance responses using a 1-9 scale, and grouped them into five groups for model fitting. However, the grouping could have been different; for instance, in grouping perceived crowding levels Neuts and Nijkamp [28] used only two categories: 1-5 (not crowded) and 6-9 (crowded), while Shelby *et al*. [29] used four categories: 1-2, 3-4, 5-7, 8-9 in a similar situation. We decided to separate the neutral category (grade 5) solely to match the concept behind neutral levels (zero acceptability) found in norm curves, and be consistent with minimum acceptable conditions [13], representing the use level when acceptance level is neutral/zero. Adding neutral response to non-disturbing level would yield higher LAD, as the probability for a visitor to give a response from 1-4 is lower than the probability to give a response from 1-5 (for any use level).

Note that, in general, model fitting requires that each category has sufficient data, while the number of categories is kept as high as possible. Also note that changing the grouping affects the LAD as defined in our paper because it affects the model fits and, therefore, the intersection of the probability densities.

Although we determine LAD as the point where visitor is equally likely to experience low and high disturbance, the LAD can also be set arbitrarily. For example, management can decide that at any given time probability of feeling disturbed at a level 7-9 should not be greater than 20%. Then, the model should consider grades 7-9 as one group, and the LAD condition is set at the point where probability density function for the group reaches 20%. Similar conditions can be set for other categories; for example, the LAD can be set such that probability of getting a disturbance grade of 1-4 is 90% or higher. In case of multiple LAD conditions, the true LAD is the one that results in the smallest acceptable use level.

### Advantages and limitations

The method presented has four major distinctive attributes, each of which can be regarded as both an advantage and a limitation. The main attribute of the method is that the measurements are preformed on-site in real conditions. The advantage is that the responses relate to actual experience, not to simulated conditions such as imagining how one would feel based on a photograph. Our method therefore eliminates the visual bias associated with sequence in which the photographs are presented to the examinees, which Gibson *et al*. [22] found to be influential. However, on-site measurements also imply that only visitors who exit the locality can be surveyed at any given time. Hence, measurements only represent the on-site conditions and visitor types. Any coping behaviors leading to spatial or temporal displacement can, therefore, introduce a bias in responses when compared to the general population: only the actual visitors at a particular use level can be interviewed.

The second major attribute is a strong link between the results and visitation patterns of the locality. A visitation pattern is a result of rather complex processes that include many variables, such as accessibility to the site, affordability of the visit, sociodemographic characteristics of the visitors, and effects of coping mechanisms that can result in spatial and/or temporal displacement(s) leading to self-selection of the surveyed visitors. The advantage of the method is that the effects of personal circumstances and coping mechanisms are an integral part of the results, i.e. the effects are included and accounted for by design. The limitation is, however, that these effects cannot be decoupled from the results; therefore, any change in visitation patterns and/or socio-demographic characteristics of visitors may require a new survey to re-parametrize the model and obtain new maximal acceptable use level.

Questionnaire shortness is the third major attribute. Including a maximum of four questions enabled fast and simple recording of visitors’ experience and, therefore, a large number and a good temporal resolution of interviews. However, lack of any demographic data limits the broadness and scope of the analysis.

Counting visitors on-site is the fourth major attribute. Depending on the choice of entry and exit points, our approach can be scaled to any area that has a common visitation pattern, from micro-localities to the whole of the PA. Such scalability is not easily achieved using the visual method; however, counting visitors on-site, especially during high-visitation times, can introduce a bias, whereas the number of visitors on a photograph is exact.

Future work could include concurrent application of both methods (the visual approach, and the approach presented in this study) on the same site. The concurrent application would enable comparison between methods, and analysis of biases inherent in each one, thus improving both.

## Conclusion

Using time-stamped short questionnaires along with visitor number counting can be a powerful tool for management in highly visited protected areas. The method presented here is simple and requires limited human and material resources, offering cost-effective monitoring and information required for proactive management. The fact that our approach was able to discern correlations between use level and disturbance, as well as disturbance level and overall satisfaction even when the maximum acceptable use level has not been reached, speaks to strengths of our approach: using the method, managers can pre-empt disturbance related to crowding, and react proactively. The approach can also be used as a tool in determining elements of the social carrying capacity [30–33].

If the results suggest LAD due to crowding has been reached, managers need not immediately reduce daily use level. Other factors could reduce disturbance below LAD. For example, crowding can be reduced by adjusting visitor movement patterns, available activities, and even just adjusting expectations by letting visitors know about crowding ahead of time. The relatively simple nature of our approach enables quick testing of the alternative strategies, thus making it possible to find the optimal way to reduce disturbance below LAD.

## Supporting information

S1 Table Survey data. The data include visitors’ responses on the three survey questions/statements, and time and location of the interview.
(XLS)
